# The Transactions of the American Medical Association

**Published:** 1850-03

**Authors:** 


### EDITORIAL DEPARTMENT.

*The Transactions of the American Medical Association,* Instituted 1847, Vol. II., Philadelphia. Printed for the Association by T. R. & P. G. Collins. 1849.

After much delay, this impatiently looked for volume has at length made its appearance. It is but justice to the publishing committee, and to the printers employed by them, to state, that the delay is in no wise attributable to neglect, or dilatoriness on the part of either. It arose from the reports, or several of them, having been retained by the chairmen of the committees for a long period, in order to prepare the manuscripts for the press, and from the necessity of sending, and waiting for the return of proofs, the authors residing in distant sections of our country. In order to obviate similar delays hereafter, the committee on publication very properly suggest that the several committees should bring their reports to the meeting complete, and legibly transcribed, so as to be handed over, at once, to the Secretaries of the Association.

In some remarks on the meeting of the Association of 1849, shortly after its occurrence, we stated that the published transactions would be found to furnish abundant evidence of the high character and usefulness of the Association. Our confidence in the correctness of this opinion is not diminished by an examination of the work. It makes a volume of *nine hundred and fifty* pages, which we feel assured will be deemed by the American medical reader to contain a vast amount of interesting and valuable matter, and will be regarded as a highly creditable addition to the medical literature of our country.

For the benefit of those of our readers who may not be acquainted with the nature and scope of the several reports, of which the volume is mainly composed, we will enumerate them, with their subjects, in the order in which they occur, giving the names of the chairmen of the committees respectively, and the number of pages which each report occupies:

*First.*—Report of the Committee on Medical Sciences, Lunsford P. Yandell, M. D., of Louisville, Ky., Chairman, 84 pages,

*Second.*—Report of the Committee on Practical Medicine, D. Francis Condie, M. D., of Philadelphia, Chairman. 57 pages.

To this Report are appended as follows:—A paper " on the more prevalent Diseases of Swedesboro, N. J., and its neighborhood, during the year 1848." By Dr. O. F. Garrison, 5 pages.

"An account of the Dysentery, as it prevailed in Cambridge, Mass., in the years 1847 and 1848." By Dr. Morrill Wyman, 6 pages.

"On Bilious Fever and Erysipelas, as they prevail in the eastern portion of New Jersey." By Dr. J. Fithian, 8 pages.

*Third.*—Report of the Committee on Surgery, Nathan R. Smith, M. D., of Baltimore, Chairman, 21 pages.

*Fourth.*—Report of the Committee on Obstetrics, C. R. Gilman, M. D., of the city of New York, acting Chairman, 21 pages.

To this Report are appended accounts of cases of retroflexion of the uteius, by Dr. A. C. Post, of the city of New York, and B. W. McCready, of the city of New York, 4 pages.

*Fifth.*—Report on Medical Education. F. Campbell Stewart, M. D., of the city of New York, Chairman, 96 pages.

To this Report are appended two papers, one by John Ware, M. D., of Boston, Mass., on behalf of the Medical Faculty of the Massachusetts Medical College; and the other by Prof. Samuel Jacskon, M. D., (chairman,) of Philadelphia. We shall refer to these papers presently. They both occupy 18 pages.

*Sixth.*—Report on Medical Literature. John P. Harrison, (lately deceased,) of Cincinnati, Chairman, 49 pages.

*Seventh.*—Report of the Committee on Publication. Isaac Hays, M. D., of Philadelphia, Chairman, 3 pages. Appended is the Report of the Treasurer, Isaac Hays, M. D.

*Eighth.*—Report of the Committee on Public Hygiene. James Wynne, of Baltimore, Chairman. To this are appended several communications from several sources, which, together with the report proper, occupy 225 pages. A large amount of statistical, and other information is therein embraced, relating to a branch of medical inquiry of an importance to the public welfare immeasurable, but which is not duly cultivated by the medical profession in general, and is very far from being appreciated by Legislators and others.

*Ninth.*—Report of the Committee on Adulterated and Sophisticated Drugs. Usher Parsons, M. D., of Providence, R. I., Chairman, 7 pages.

*Tenth.*—Report of the Committee on Indigenous Medical Botany. N. S. Davis, M., D. of Chicago, Ills., Chairman, 14 pages.

To this Report are appended a communication on the medicinal plants indigenous in South Carolina, by Francis P. Porcher, M. D., 87 pages; and a communication on the indigenous medical botany of Massachusetts, by Stephen W. Williams, M. D., 65 pages.

The foregoing schedule will enable the reader to form an idea of the contents of the volume, and of the relative space devoted to the several departments of scientific investigation. The reputation of the authors of the Reports will constitute a basis for some pre-estimation of the manner in which the various subjects are treated. The magnitude of the work is a sufficient obstacle in the way of undertaking any thing like an analytical review of the numerous papers of which it is composed, But this is not called for, if ever so practicable, inasmuch as it is to be hoped, and presumed, that the work itself will have a wide circulation among members of the medical profession of this country.

It is hardly necessary to say, it is by no means to be inferred from the tenor of the foregoing remarks, that we deem the volume of Transactions devoid of faults and defects. We do not, however, design, at this time, to enter upon an inquiry respecting the various points of view in which the work offers an opportunity for criticism, nor, on the other hand, to engage in the more agreeable task of pointing out its particular excellencies. But we should do injustice to our sentiments, we will not say as a journalist, but as a member of the medical profession, if we omitted to say that in a hearty commendation of the volume, as a whole, we would not be considered to include one of the two papers appended to the Report on the subject of Medical Education. We refer to the report of the Special Committee appointed to prepare "a statement of the facts and arguments which may be adduced in favor of the prolongation of the courses of medieal lectures to six months." The occasion giving rise to this report should be mentioned. Dr. Ware having submitted an able paper, on behalf of the Medical Faculty of the Massachusetts Medical College, advocating the propriety of limiting College terms to four months, its publication, after some discussion, was ordered by the Association. It was then resolved that a committee be appointed to prepare, as a counter report, a statement of the *facts* and *arguments* in favor of six months' terms. The latter report was written subsequently to the meeting of the Association, and, consequently, could not receive its sanction or adoption. It is but due to the Association that this be borne in mind by the readers of the Transactions. The Association can not be held responsible for the character of that report, notwithstanding the assumption by the committee to speak in behalf of the Association. The responsibility, under the circumstances, obviously rests solely with the Committee.

We confess that we have perused the report referred to, with warmer feelings than are expressed by the terms disappointment and regret. The charges and insinuations directed toward the Medical Profession of this country, and toward American Medical Schools, which it contains, we cannot but deem unmerited and libelous, and when it is considered that they form a portion of the transactions of our national association, which are to circulate extensively, not only at home, but abroad, it requires no small degree of forbearance to repress other emotions than deep mortification. In perusing the report, we had marked several passages for quotation, but we choose rather to refer the reader to the entire report. It is bad enough, and sufficiently calculated to test the power of endurance of the medical profession, to be calumniated by foreigners, and vilified by the infatuated partizans of medical delusions which surround us, but when our own brethren and countrymen, and those, too, occupying eminent positions, denounce the profession of this country as ignorant and degraded, declaring, in unqualified terms, that medical science is not taught in our schools, that we have no claim to any active participation in the movement of progress, that new branches constituting the scientific basis of medicine are unknown, even by name, to large numbers of our practitioners, etc., etc., etc., what can we expect either from captious brethren at a distance, or open enemies nigh at hand? We complain not a little of the want of respect for, and confidence in the profession of medicine and medical science, by the public, but, how can we, consistently, utter this complaint while the worst that can possibly said of us by the bitterest of our opponents, is echoed from our own ranks! What claim can we urge npon the public for consideration and support, while prominent members of the profession renounce every claim to self-respect! It seems to us that it is time for the American medical profession to awake to a proper appreciation of the injustice heaped upon it by its own members, and to rebuke the slanderous imputations which have become so common with some medical writers. On this subject we would commend to the attention of the reader, the admirable remarks by Prof. Coventry, which are contained in the article in the original department of the present number of this Journal, on self-reformation in medicine. If we do not err, our friend and colleague has directed attention in that article, to one of the prime sources of the dissatisfaction which many members of the profession of this country appear to take no small degree of pleasure in indulging.

We have expressed, in general terms, our sense of the report of the special committee appointed to prepare a statement of facts and arguments in favor of the prolongation of the courses of medical lectures to six months." We do not propose, in this connection, to engage in a critical examination of the report. The topics which it involves we shall take occasion to consider, from time to time, without farther reference to the report. The report, indeed, will hardly bear critical analysis. The committee were appointed to present *facts* and *arguments*, but in the place of facts we find unproved statements, and bold assertions are put forth in lieu of arguments. The reader has only to deny the statements, and protest against the assertions, and the subject remains precisely in the position which it occupied prior to the discussion.

We desire it to be understood that in making these comments we have no reference whatever to the point at issue between the Faculty of the Massachusetts Medical College, and the Committee appointed to make a counter report—in other words, to the prolongation of courses of medical lectures to six months. The question of four or six months' terms is extrinsic to the features in the latter report which appear to us so unjust, and abusive as regards the medical profession and medical schools. It is the spirit of the report, and its untenantable aspersions to which we take exceptions. Personally, we are not opposed to an extension of Lecture terms to six months. We were among the first to advocate this measure to the extent of our small ability, and we concurred most heartily in its adoption by the Institution with which we are connected. Experience of its results has led us, with others, to entertain doubts whether it be the best method that can be devised to provide for a larger amount of instruction than can be given in courses of four months. Still, if it be the general opinion of the profession that this is the best plan, we stand ready to do what we can to promote its general adoption. This topic we reserve for further discussion at a future time. We have alluded to it simply to disclaim any settled conviction with regard to it.

In closing this notice of the "Transactions," we would state, for the information of those of our readers who are not members of the Association, and who wish to procure the work, that orders will be received by Messrs. Lea and Blanchard, Philadelphia. The price, per copy, in paper covers, is $3,00; in muslin, $3,50. Societies which have been represented in the Association, will be furnished copies for their members on the following terms:—three copies in paper covers for $5,00; three copies of vols. one and two $8,00. The amount must be remitted to Isaac Hays, M. D. Treasurer of the Association, in par funds. Delegates having paid $3,00 are entitled to one copy, and those who have paid $5,00 are entitled to three copies in paper covers. Permanent members are entitled to one copy on remitting $2,00, and to three copies for $5,00.

The following critical notice of one of the Reports contained in the Transactions, has been furnished by an esteemed correspondent:

To the Editor of the Buffalo Medical Journal.

Dear Sir—

In looking over the very elaborate "Report," of the Committee on Medical Education, under the head of Legal Requirements of Medical practitioners in the several states, I was surprised to find the following: "New York, no restriction since 1844, when the law was repealed: all persons have the right to practice and recover compensation for their services"; and this short paragraph of two and a half-lines is all the notice bestowed on the law regulating the profession in the state of "New York"—the Chairman's own state,—whilst more than four pages are devoted to the regulations in Europe, which are of no practical importance, and which have little or no interest for the profession in this country. The paragraph —the whole of which I have quoted—is calculated, and no doubt intended to convey the impression that we have no law regulating the profession in this state. Whether this was the result of ignorance, or intentional, is impossible to be gathered from the wording of the paragraph. But what are the facts? The laws regulating the practice of medicine were drawn up with much care, and mature deliberation—and after consultation with some of the leading men in the profession, it has since undergone some slight modifications, but is substantially the same as it came from the hands of the revisers, and is probably as stringent as those of any state in the union. The article of 1844, to which the paragraph alludes, as will be seen, repealed only a single section, which applied to persons out of the profession, not to members of the profession, and was considered by the intelligent portion of the profession, who knew what the law was, a decided improvement. As originally reported by the revisers, the practicing of medicine without a license was made a misdemeanor, punishable by fine and imprisonment; this law, however, was permitted to stand but a short time on the statute-book. It was soon repealed, and the old law on the subject re-enacted.

This forbids any person practising medicine without a license under a penalty of $20, but declared that it should not apply to any person practising with roots and herbs the growth of this country. This exception—to say nothing of the absurdity of the distinction—completely nullified the whole section. Every person who chose, practised with impunity under this clause, as they could not, like the regular practitioner, be held liable for damages resulting from their ignorance. The Legislature of 1844 wisely determined that this anomalous section should no longer disgrace the laws of the state; leaving the law regulating the medical profession, where it was—with all its restrictions and guards,—but permitting any persons not of the profession to prescribe and practice, if any one chose to employ them, and at the same time holding them responsible for their acts—not merely for damages to the parties injured—but for practicing a fraud upon community in professing to understand that of which they were ignorant.

The limits of this paper will not admit of our giving a detail of the whole law on this subject; a brief outline, together with the act of 1844, will enable your readers to know what are the main provisions of the law at the present time. The law evidently recognizes only licensed physicians and surgeons as being of the profession.

The physicians and surgeons of each county are authorized to assemble at some place, of which due notice shall be given, and organize themselves into a society for that county. The several societies have, for the purposes specified in the act, all the powers of corporate bodies, they can sue and be sued, form their own By-laws and make all other regulations, not inconsistent with the laws of this State or of the United States, which appertain to the purposes of their organization. By section vii, chap. xiv, part i, R. S. it in directed that the President of each Medical Society shall give personal notice to every practitioner in said county not being a member of said society, to apply within 60 days for admission into the society, and in case of neglect to do so to forfeit his license.

Another section declares that the regular term of study shall be four years, but one year may be deducted if the student has, after the age of 16, devoted one or more years to the study of those subjects usually pursued in literary colleges, or shall have attended one full course of medical lectures given in a regularly incorporated medical college.

Every physician, on receiving a student into his office, is requested to file a certificate of the fact in the office of the President of the County Society, and if it is determined to make any deduction from the period of four years, to state it in the certificate.

The censors of the county society, before examining a student, must have satisfactory evidence that he is 21 years of age, of good moral character, and have complied with the requirements of the law as to the term of study. The student must be examined in the county where he has pursued his studies.

A student having been rejected in one county, cannot apply to the censors of another county, but may appeal to the censors of the State Medical Society. Physicians or surgeons coming from a foreign country are required to be examined by the state censors. Physicians and surgeons coming from another state must furnish satisfactory evidence of their qualifications to the censors of the county society, and file a copy of their diploma. Every physician and surgeon is required to file a copy of his diploma or license in the clerk's office of the county where he resides. The regents of the University, and the several Medical Colleges, to grant the degree of M. D., must, in addition to the above, have evidence that candidates have attended two full courses of medical lectures, the last of which must have been at the institution granting the diploma. It will be seen from the above, that with the exception of a few specific regulations, as the age, the time of study, and requirement of a good moral character, the whole regulation of the profession is left to its own members, who are clothed with the most ample authority by the legislature.

The following is the act of 1844, and the profession can judge how far it justifies the impressions produced by the paragraph in the Transactions above quoted.

The 22d sec. of chap. xiv, title vii, part 1 of the revised statutes, and all laws of this state which prohibits any person from recovering, by suit or action, any debt, or demand arising from the practice of physic or surgery, or compensation for services rendered in attending the sick or prescribing for them, is hereby repealed." R. S. part 1, chap. 14th, sec. 24.

"No person shall be liable to any criminal prosecution, or to any indictment for practicing physic and surgery without licence, except in cases of mal-practice, gross ignorance, or immoral conduct in such practice." Sec. 24.

All and every person, not being a licensed physician, who shall practice or attempt to practice physic or surgery, or who shall prescribe for, or administer medicine, or specifics to, or for the sick, shall be liable for damages in case of mal practice, as if such person was duly licensed to practice physic and surgery. Any person, not being a licensed physician, who shall practice or profess to practice physic or surgery, or shall prescribe medicines or specifics for the sick, and shall in any court having cognizance thereof be convicted of mal-practice, gross ignorance, or immoral conduct, shall be deemed guilty of a misdemeanor and liable to a fine of not less than fifty, and not exceeding one thousand dollars, or imprisonment in the county jail not less than one month or exceeding twelve months, or both, at the direction of the court. R. S. sections 24, 25, 26, and 27, of part 1, chapter 14.

On looking over the portion of the Report devoted to Medical Education, I was sorry to find the same apparent ignorance of the system of Medical Education in our own country, and the same admiration of every thing that is foreign. The two systems are entirely different, every one will admit, but to infer that the one is so much superior to the other, as is done in the report, is an assumption which is entirely gratuitous. In Europe the student is expected to acquire his profession at the schools and hospitals, and the laws are framed accordingly. In this country the student is expected to acquire his profession in the office of his private preceptor, and the attendance on lectures is merely accessory. This is evident from the tenor of the law, that—although it recommends attendance on lectures—does not make it indispensable. Much is said on the subject of clinical instruction, and it is constantly inferred, if not stated in so many words, that our sudents in the country have no clinical instruction. Whereas, in fact, the whole pupilage with our intelligent practitioners, is one course of clinical instruction, as much superior to merely following a visiting physician through the wards of a hospital, as usually conducted, as the moral and physical purity of a healthy country village is superior to the sinks and stews of a large city. I have examined this subject thoroughly, both in Europe and in this country, and have no hesitation in asserting that the combined system of private instruction ,with public lectures, is better adapted to the condition and wants of the country, better for the morals, better for the health of the student, much less expensive; and taking the same students, will make better citizens, and better practitioners, than the system adopted either in London or Paris, admitting, of course, that the same length of time is devoted to the study in each. C. B. C.

